# Inhibitory and preventive effects of *Arnebia euchroma* (Royle) Johnst. root extract on *Streptococcus mutans* and dental caries in rats

**DOI:** 10.1038/s41405-024-00196-6

**Published:** 2024-03-02

**Authors:** Zeyu Wu, Jie Song, Yangyang Zhang, Xiyu Yuan, Jin Zhao

**Affiliations:** 1 Department of Cariology and Endodontics, The First Affiliated Hospital of Xinjiang Medical University (The Affiliated Stomatology Hospital of Xinjiang Medical University), No. 137 South Liyushan Road, Urumqi, 830054 People’s Republic of China; 2 Stomatology Disease Institute of Xinjiang Uyghur Autonomous Region, No.137 South Liyushan Road, Urumqi, 830054 People’s Republic of China

**Keywords:** Streptococcus mutans, Antibiotic prophylaxis in dentistry

## Abstract

**Background:**

Dental caries is one of the prevalent conditions that threaten oral health. *Arnebia euchroma* (Royle) Johnst. root (AR) extracts exhibit anti-inflammatory, anti-cancer, and antibacterial properties. This study was designed to investigate the antibacterial impact of AR extract on *Streptococcus mutans* (*S. mutans*) UA159 and the anti-caries effect on rats.

**Methods:**

The antibacterial activity of AR extract against *S. mutans* and its biofilm was determined using the bacterial sensitivity test, the biofilm sensitivity test, and the live-dead staining technique. By fluorescently tagging bacteria, the influence of bacterial adhesion rate was determined. Using a rat caries model, the anti-caries efficacy and safety of AR extract were exhaustively investigated in vivo.

**Results:**

AR extract inhibit not only the growth of *S. mutans*, but also the generation of *S. mutans* biofilm, hence destroying and eliminating the biofilm. Moreover, AR extract were able to inhibit *S. mutans*’ adherence to saliva-encapsulated hydroxyapatite (HAP). Further, in a rat model of caries, the AR extract is able to greatly reduce the incidence and severity of caries lesions on the smooth surface and pit and fissure of rat molars, while exhibiting excellent biosafety.

**Conclusions:**

AR extract exhibit strong antibacterial activity against *S. mutans* and can lower the incidence and severity of dental cavities in rats. These findings suggest that *Arnebia euchroma* (Royle) Johnst. could be utilized for the prevention and treatment of dental caries.

## Background

Caries is a chronic infectious disease mediated by bacterial biofilms, driven by sucrose, and influenced by various variables [1]. Currently, dental caries is the third most prevalent noncommunicable disease in the world, after cancer and cardiovascular disease. Permanent teeth caries and deciduous tooth caries were second and fifth, respectively, among the 10 most prevalent diseases in the globe according to an epidemiological survey conducted in 2016 [2]. Dental caries is still an extremely prevalent disease in the oral cavity, with rates of 62.5% for deciduous teeth in children ages 3–5 and 41.9% for permanent teeth in children ages 12 to 15, according to the 4th National Oral Health Survey conducted in the Mainland of China [3]. According to incomplete data, there are approximately 700 different types of bacteria in the oral microbial community, and they play a crucial role in maintaining dental health [4].

Among them, *Streptococcus mutans* (*S. mutans*) is recognized as the main cariogenic bacteria [5, 6]. It metabolizes sucrose and also synthesizes water-insoluble glucans, which are essential for the formation of stable biofilm matrix. It also produces the biofilm matrix that promotes bacterial adhesion and colonization on tooth surfaces while maintaining an acidic environment for the growth of cariogenic bacteria. Therefore, selective *S. mutans* in complex dental plaque biofilms, reflecting the anti-caries efficacy of drugs by suppressing the growth of *S. mutans* and its virulence factors, may be a promising method for preventing dental caries.

With an in-depth understanding of the etiology and pathogenesis of dental caries, fluoride [7], chlorhexidine (CHX), and other drugs have been developed and put into caries control one after another, among which CHX is often made into mouthwash because of its remarkable antibacterial ability [8]. In addition, researchers often use CHX at 0.12% concentration as a gold standard to assess the anti-cariogenic activity of the agent to be tested [9]. However, along with the overuse of CHX [10], side effects such as oral microecological disorders, drug-resistant bacteria, or fungal overgrowth have occurred one after another.

Consequently, ecological caries prevention approaches are increasingly coming into view, and it is required to create novel anti-caries strategies based on ecological prevention and to encourage the creation of a healthy and stable oral biofilm environment [11]. Existing ecological anti-cause approaches include antimicrobial peptides, probiotics, natural compounds, etc. [12]. Natural products have become the focus of research on caries prevention and treatment due to their abundant sources, low cost, potent antibacterial properties and good biological safety and other characteristics [13].

*Arnebia euchroma* (Royle) Johnst. (Xinjiang-Zicao or Ruan-Zicao in Chinese) is a commonly prescribed traditional herbal medicine in Xinjiang, Tibet, and Mongolia and was first recorded in “Shen Nong’s Materia Medica” [14]. *Arnebia euchroma* (Royle) Johnst. is mainly distributed in high-altitude areas in Central Asia, North Africa, Afghanistan, and the western Himalayas [15]. *Arnebia euchroma* (Royle) Johnst., according to “ The Pharmacopoeia of the People’s Republic of China 2020,” has the ability to detoxify the body, eliminate heat from the body, and cool the blood. Both Uygur and Traditional Chinese medicine make extensive use of it [16]. Numerous bioactive substances found in *Arnebia euchroma* (Royle) Johnst. [17], such as naphthoquinones, phenolic acids, and alkaloids, have been shown to have antibacterial, anti-inflammatory, antioxidant, and other properties [15, 18, 19]. Based on our previous research, *Arnebia euchroma* (Royle) Johnst. can inhibit the growth, acid generation, and sugar production of the major cariogenic bacteria [20], but its efficacy and safety in preventing caries against *S. mutans* adhesion and in vivo caries have not been investigated.

In this research, the effects of AR extract on the growth and adhesion of *S. mutans* and their biofilm were assessed in vitro. To give theoretical and experimental support for the development of AR extract as an anti-caries agent, the extract’s safety and effectiveness against *S. mutans* were assessed in a rat caries model.

## Materials and methods

### Ethics statement

The “Ethics Review Committee of Xinjiang Medical University” gave its clearance for this research’s conduct (IACUC-JIPD-2019030). The China Ministry of Science and Technology’s Guide for the Care and Use of Laboratory Animals was followed in all experiments.

### Bacterial strain and growth conditions

*S. mutans* UA159 (ATCC 700610, a cariogenic bacterial pathogen) provided by the Guangdong Microbial Culture Collection Center (GDMCC) was incubated in brain heart infusion (BHI; Oxoid, UK) broth containing 1% sucrose and incubated for 24 h at 37 °C. After incubation, the bacterial concentration was 107 colony forming units (CFU)/mL ([Optical Density (OD) 630 nm = 0.2)] as determined by spectrophotometry. The strains were preserved and stored in 20% glycerol at −80 °C for an extended period of time.

### Plant material and chemical reagents

Dried roots of *Arnebia euchroma* (Royle) Johnst. were purchased from the local market of Urumqi and the species was identified and authenticated in Department of Pharmacology, Xinjiang Medical University, China. The dried roots of *Arnebia euchroma* (Royle) Johnst. were dried at 25 °C and ground to a fine powder in a grinder, passed through a 110-mesh stainless steel screen and stored in a desiccator. First, 500 g dried roots of *Arnebia euchroma* (Royle) Johnst. powder was extracted by soaking in 95% ethanol (w/v, 1:10) at room temperature and repeated three times. The extract was concentrated by evaporation under vacuum (50 °C) in a rotary evaporator and was freeze-dried. Samples were collected and stored in amber bottles at −4 °C [21].

The ingredients of AR extract were determined by Tandem Mass Spectrometry (MS/MS), in combination with an Ultra Performance Liquid Chromatography (HPLC Thermo UltiMate 3000, Thermos Fisher Scientific, Waltham, MA, USA) and a mass spectrograph (AB 5600+ Triple TOF, SCIEX, Framingham MA, USA). The collected data was initially processed and confirmed according to the exact Mass (mass error ≤30ppm), and then, Secondary mass spectrometry database (Massbank, GNPS, RIKEN PlaSMA, BMDMS-NP, mzClound, etc) was used for substance identification and analysis of the collected data. The results showed that AR extract mainly contain alkaloid, Flavonoid, phenol, coumarins, terpenoids, etc. Please refer to the appendix Table S1 for the further details.

### Determination of minimal inhibitory concentrations (MIC) and minimum bactericidal concentration (MBC)

Refer to the method of Preparing Dilutions of Water-Insoluble Antimicrobial Agents to Be Used in Broth Dilution Susceptibility Tests published by the Clinical and Laboratory Standards Institute in 2022 for the determination of MIC and MBC [22]. In a nutshell, fresh BHI was used to dilute the collected *S. mutans* cultures into 96-well plates (NEST, Wuxi, China) at 1.0 × 10^7^ CFU/mL. The AR extracts were diluted with BHI medium and dimethyl sulfoxide (DMSO) with reference to the double dilution method, and the final concentrations of the extracts evaluated ranged from 32 to 0.25 mg/mL. This was done to establish MIC values of AR extract for animal research and clinical study direction. DMSO with a final concentration of 1% used in the experiment has been proved to be non-toxic to bacteria [23]. Bacteria with 0.12% CHX (Yuanye, Shanghai, China) treatment were utilized as positive controls, whereas untreated bacteria and BHI broth medium were used as negative controls. The 96-well plates were grown on a rotary shaker (150 rpm) at 37 °C and the absorbance of each well at a wavelength of 595 nm was measured after 24 h using a Multiskan Spectrum (Thermo Fisher Scientific, Inc.) and the growth inhibition rate was calculated according to the formula (growth inhibition rate = [1-(A_595_ experimental group / A_595_ negative control group)] × 100%) [24]. MIC_50_ was established as the medication concentration that 50% inhibited growth.

The BHI agar dilution method was used to calculate the MBC. Bacterial cultures from wells with test samples having concentrations equal to or higher than MIC_50_ were transferred to BHI agar plates and incubated for 24 h. MBC was defined as the lowest concentration at which there were no visible bacterial colonies on the agar plates after 24 h of incubation. The assays were performed in triplicate on three different occasions.

### Determination of minimum biofilm inhibition concentration (MBIC_50_) and minimum biofilm reduction concentration (MBRC_50_)

MBIC_50_ and MBRC_50_ examined the effect of AR extract on the formation and reduction of *S. mutans* biofilm. The method for determining MBIC_50_ and MBRC_50_ was performed as previously described [24, 25], with some modifications. MBIC_50_ was defined as the lowest concentration of agent showing 50% or more inhibition of biofilm formation. Briefly, the bacteria were grown in BHI broth containing 1% (w/v) sucrose [26]. With a final bacterial concentration of 1.0 × 10^7^ CFU/mL as required for bacterial susceptibility testing; the AR extract concentrations varied from 32 to 0.25 mg/mL. The experimental groupings were consistent with those of MIC and MBC. The supernatant medium was carefully collected after the 24 h incubation period at 37 °C for static incubation, and the planktonic bacteria were then eliminated by thoroughly washing each well three times in 200 μL of PBS. The wells were stained with 0.1% (w/v) crystal violet for 5 min after being fixed with methanol for 15 min. The excess dye was removed, the plate was washed with water, and air-dried for 1 h. The 96-well plate was then shaken for 30 min at room temperature after 200 μL of 95% ethanol had been poured to each well. To calculate the biomass of the biofilm, the OD_595_ nm was measured using Multiskan Spectrum.

The MBRC_50_ was defined as the lowest concentration of agent that reduces the formed biofilm by 50% or more. Briefly, bacteria were grown in BHI broth containing 1% (w/v) sucrose. The bacteria were inoculated at a final concentration of 1.0 × 10^7^ CFU/mL, and after 24 h of static incubation at 37 °C, the planktonic cells were removed by decanting the medium and the wells were gently washed with PBS. The AR extract concentration ranged from 32 to 0.25 mg/mL and was added to the above formed bacterial biofilm. The rest of the steps followed the same procedures as in the MBIC_50_ described above.

Bacteria with 0.12% CHX treatment were utilized as positive controls, whereas untreated bacteria and BHI broth medium were used as negative controls. The assays were performed in triplicate on three different occasions.

### Live/dead bacteria staining

Bacterial viability was determined using the Dojindo Bacterial Viability Detection Kit-DAPI/PI (Kumamoto, Japan) [27]. According to the manufacturer’s recommendations, *S. mutans* was cultured on sterilized glass discs and allowed to grow for 24 h in BHI medium to produce biofilms. Planktonic bacteria were removed using 37 °C PBS after biofilm formation, and AR extract was then formulated into interventions at 8, 4, and 2 mg/mL as experimental groups, 0.12% CHX interventions as positive control groups, BHI medium interventions as blank control groups, and 1 percent DMSO interventions as solvent groups. The cultures were gently removed after 24 h of anaerobic incubation at 37 °C, washed twice with sterile water, stained with Bacstain-Bacterial Viability Detection Kit-DAPI/PI, and then left to sit at that temperature for 15 min in the dark. Under a fluorescence microscope, stained bacterial biofilms were viewed (Leica Mannheim; Wetzlar, Hessen, Germany). All bacteria can be stained with DAPI, which is membrane permeable and identifiable by blue fluorescence. Damaged bacterial membranes are stained with red fluorescence by PI, which is membrane impermeable. For each sample, three fields of vision were chosen at random under a 100× objective lens. Based on examination of the ratio between the coverage of live bacteria to total bacteria using the software Image J (National Institutes of Health, Bethesda, MD, USA).

### Adherence to hydroxyapatite (HAP)

**Fig. 1:**
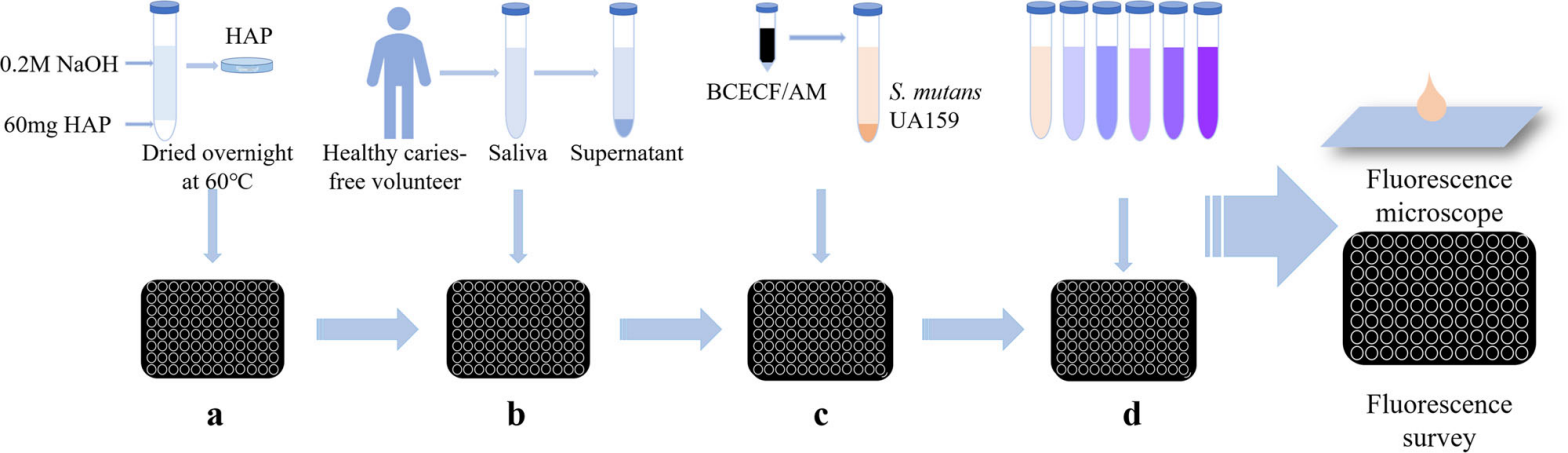
Flow chart of adhesion experiment. Experimental procedure for evaluating the effect of AR extract on the adhesion properties of S. mutans by saliva-coated HAP. The experiments included: (a) HAP 96-well plate fabrication; (b) saliva-coated HAP; (c) loading fluorescent probes to S. mutans; (d) Testing AR extract’s ability on the adhesion of S. mutans carrying fluorescent probes in saliva-coated HAP; the final step was to observe the adhesion of S. mutans using a fluorescence microscope.

To simulate in vitro the inhibitory effect of *S. mutans* adhesion to the main component of dental enamel, HAP, within the oral microenvironment, we referenced the experimental method previously used [28] and made certain improvements. We used fluorescent probes to label *S. mutans*, which were then co-incubated with saliva and AR extract for a set period. Subsequently, the unattached bacteria from each group were removed, and the *S. mutans* adhered to the HAP surface were resuspended. The inhibitory effect of AR extract on the adhesion of *S. mutans* was assessed by measuring the fluorescence intensity of the remaining bacteria. This method allowed us to evaluate the extent of inhibition of the AR extract on the *S. mutans* adhesion process. Non-stimulated saliva was collected from the specific volunteer (without systemic and oral diseases) after 4 h of feeding as approved by the Ethics Review Committee of Xinjiang Medical University (IACUC-JIPD-2019030). Volunteers were asked to read the completed consent form and sign it if they agreed with its contents. Saliva samples (40 mL) were collected and centrifuged at 15000 rpm for 20 min at 4 °C before being filtered through a 0.22 μm sterile filter (Millipore Corp, Billerica, MA, USA) and stored at 4 °C. According to the steps outlined by Shahzad et al. [29], the 96-well black-walled, clear-bottomed microplates with HAP coating were created. Prepare a homogenous HAP suspension by dissolving HAP in sterile saliva (5% w/v), shaking it well, and then pouring it into the well plates. The plate was then agitated for 15 min at 37 °C and 200 rpm, dried, and then given two PBS washes. *S. mutans* was labeled with 2’,7’-Bis-(2-Carboxyethyl)−5-(and-6)-Carboxyfluorescein, Acetoxymethyl Ester (BCECF/AM, Beyotime, Shanghai, China). BCECF/AM-labeled *S. mutans* (100 µL) was added to 96-well plates along with AR extract at concentrations ranging from 4.0 to 0.25 mg/mL. The 96-well plate was incubated in the dark at 37 °C with low-speed shaking for an additional 2 h. Unbound bacteria were aspirated and washed twice with PBS. The relative fluorescence units (RFU; excitation wavelength of 495 nm; emission wavelength of 525 nm) corresponding to the level of bacterial adhesion were determined using the Victor Nivo Multimode Microplate Reader (PerkinElmer, USA). Control wells without the test drug were used to determine 100% adhesion values, while wells without bacteria were used as controls to determine basal autofluorescence. All these determinations represent the average of three independent experiments. At the same time, the adhesion ability of *S. mutans* to HAP was observed under a fluorescence microscope (Leica Mannheim; Wetzlar, Hessen, Germany) on the above-mentioned plates. Five fields of view were randomly selected for each sample under a 100× objective lens. A flow chart of the adherence to HAP experiment is shown in Fig. 1.

### In vivo efficacy of AR extract

#### Rat caries model

**Fig. 2:**
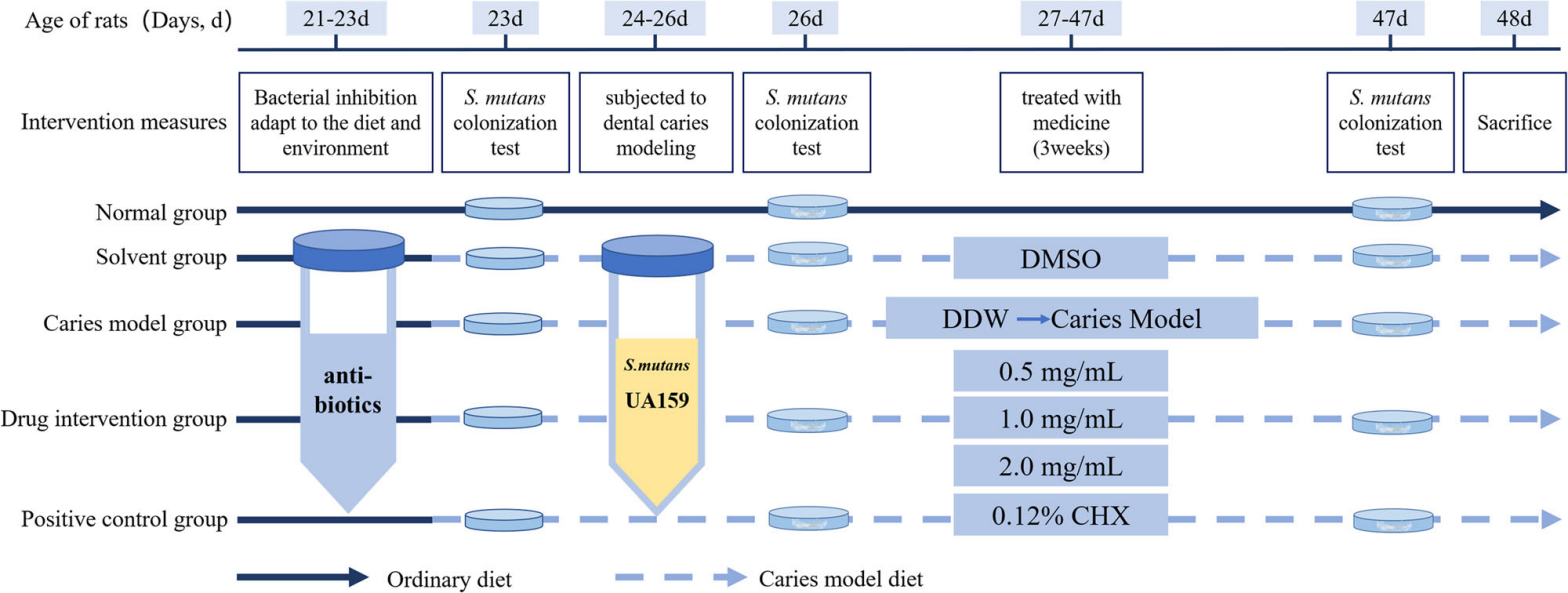
Flow chart of rat caries experiment. The procedure for the study of the anti-caries effect of AR extract on rat with dental caries in vivo, mainly includes: inhibition of oral endogenous bacteria in rats, inoculation of S. mutans, and periodic experimental drug intervention. (DMSO: dimethyl sulfoxide; DDW: double distilled water).

In total, 42 specific pathogen-free (SPF-SD) 21-day-old SD rats (only males) were purchased at the Animal Experiment Center of Xinjiang Medical University and the animal experiments were performed using the previously described methods [30]. First, to avoid the effect of endogenous oral microorganisms, 21-day-old SD rats were fed antibiotic water containing benzylpenicillin (200 μg/mL) and streptomycin sulfate (1500 μg/mL) and a diet supplemented with antibiotics (1 g/kg) for three consecutive days. Saliva from SD rats was inoculated onto Mitis Salivarius Agar Base (MSA, OXIOD, UK) to examine the therapeutic efficiency of oral endogenous antibacterial [31]. The above SD rats were randomly divided into seven groups (*n* = 6 for each), as reported below: (1) 0.5 mg/mL AR extract; (2) 1 mg/mL AR extract; (3) 2 mg/mL AR extract; (4) 0.12% CHX (positive control group); (5) 1% DMSO (solvent control group); (6) double distilled water (DDW, negative control group); and (7) no intervention (blank control group). For the next three consecutive days, *S. mutans* UA159 suspension (500 μL, 1 × 10^8^ CFU/mL) was inoculated onto the teeth of each rat (twice daily). Each rat was fasted for 0.5 h before being inoculation and after bacterial inoculation. Then, the efficiency of bacterial inoculation was examined as described above. The above drug solution will be prepared and transferred to Eppendorf tubes (1 mL/tube), and each rat will be used independently. Sterile cotton swabs were dipped into the above solution and used to topically clean the molars and oral mucosa of the rats. The residual drug solution was then used to rinse the rats’ mouths. Water and food were abstained from for 0.5 h after treatment. Treatments were performed daily at 9 am and 9 pm for 3 weeks by the same operator. During 27–47 days, rats were fed ad libitum a cariogenic Keyes 2000 diet (Xietong Organis, Jiangsu, China) and 5% sucrose water. The body weight of the rats was also continuously recorded for 27–47 days to monitor for signs of drug toxicity [32]. A flow chart of group allocation is shown in Fig. 2.

#### Bacteriological examination of saliva

On day 47, 0.2% pilocarpine nitrate (0.4 mL/100 g) (bauschlomb, Shandong, China) was injected intraperitoneally, and 100 μL of saliva was collected from each rat and inoculated onto MSA for colony counting [33].

#### Keyes scoring and X-Ray

At the end of the experiment (48 days), the rats were anesthetized by Zoletil® 50 (active ingredients: tiletamine 125 mg, zolazepam 125 mg, excipients; produced by Virbac Co., France) and and Lumianning II (a new compound preparation composed of xylazine hydrochloride and other optimally proportioned drugs, manufactured by Jilin Huamu Animal Health Products Co., Ltd., approval number: Veterinary Drug (2009)070011582, production batch number: 140610) and euthanized. After decapitation of the rat, the maxilla and mandible of the rat were removed and placed in an autoclave at 121 °C for 15 min. The attached soft tissue was peeled off with a scalpel, and the molars were cleaned and dried at room temperature. Radiographic analyses were performed using an X-ray machine (Intra Oral X-ray Unit INTR, Nahkelantie 160, Finland). The X-ray tube was operated at 60 kV, 7 mA for 0.1 s, and the source-to-sensor distance was 10 cm. After photographing, all specimens were immersed in a 0.4% ammonium salt solution (Yuanye, Shanghai, China) for staining for 16 h, and protected from light. Finally, the carious areas were stained red by observation through a stereomicroscope (Leica Mannheim; Wetzlar, Hessen, Germany), and the caries condition of each rat was assessed using the Keyes’ scoring method. The smooth and occlusal surfaces of caries and their severity (E, enamel only; Ds, dentin exposed; Dm, 3/4 of affected dentin; Dx, all dentin affected) [34].

#### CLSM tested Dental tissue grinding plate

The rat molars were sawed sagittal with diamond wire saw and made into Dental tissue grinding plates about 300 μm thick under running water. The grinding pieces were immersed in 0.1 mmol/L Rhodamine B solution, sheltered from light, and soaked at 37 °C for 24 h. After dyeing, rinsed the excess pigment on the surface of the samples with running water and wiped dry. The samples were placed in the center of the slide, sealed with the sealing solution (Glycerin: PBS = 1:1), placed coverslip on the glass slide gently, and then observed under CLSM. Imaging principle of this experiment: In the early stage of caries, tooth demineralization leads to enlarged pores on the enamel surface, and CLSM can detect the fluorescence emitted by the fluorescent dye entering the pores [35]. The stronger the fluorescence intensity, the more serious the demineralization. The larger the fluorescence range, the more serious the caries. The continuity and integrity of enamel or dentin can also be observed. Images were analyzed using ImageJ pro plus software.

#### Drug safety evaluation

After the execution of the rats, the rat oral mucosa was collected and stained for H&E, while the heart, liver, spleen, lung, and kidney were harvested and weighed to evaluate the biocompatibility of the different treatments.

#### Statistical analysis

Statistical analysis was performed using SPSS 26.0 software (IBM Corporation, Armonk, NY, USA). Data were visualized using GraphPad Prism 8.0 (GraphPad Software, La Jolla, CA, USA) software. The experimental data were tested for normality and homogeneity of variance. If the data met normality and homogeneity of variance, one-way ANOVA was used, and the LSD test was used for pairwise comparison. Pairwise comparisons were made using the Kruskal-Wallis Non-Parametric Hypothesis Test for non-normally distributed data. *P* < 0.05 was set to be statistically significant. The Image J (free and open-source software for scientific image analysis) software was used to quantify the fluorescence intensity.

## Results

### AR extract qualitative analysis

There has been a lot of research on the application of MS/MS in the identification of plant extracts [36]. In the chromatogram of AR extract (Fig. S1), Both positive and negative ionization chromatograms show a rich concentration of the majority compounds between 0.5 and 29 min. The primary components of *Arnebia euchroma* (Royle) Johnst. root extract that are potentially capable of exerting antimicrobial effects are the Shikonin, Acetylshikonin, beta,beta-Dimethylacrylshikonin, Isovalerylshikonin, Deoxyshikonin, Arnebinone, Stigmasterol and coumarins, etc. (Table S1, S2). The main compound classification within the extract is shown in Fig. S2.

### AR extract inhibited the growth of *S. mutans* and its biofilm

We found that AR extract had a certain inhibitory effect on the growth of *S. mutans*. More specifically, the AR extract was able to inhibit the growth of 50% of the planktonic *S. mutans* at a concentration of 1.0 mg/mL, and the MBC value was 8.0 mg/mL measured by agar dilution method, as shown in Table 1. We examined the impact of AR extract on the development of *S. mutans* biofilms because cariogenic biofilms are crucial in the emergence of dental caries. As shown in Table 1, we measured the MBIC_50_ and MBRC_50_ of AR extract on *S. mutans* biofilms at 4 mg/mL and 8 mg/mL. All the above results showed that the AR extract not only has an inhibitory effect on the growth of planktonic bacteria of *S. mutans* but also inhibits the formation of biofilms and clears some of the formed biofilms. Meanwhile, bacterial growth was unaffected by 1% DMSO (solvent control). In this experiment, 0.12% CHX can completely inhibit the growth of planktonic bacteria and biofilm of *S. mutans*.

**Table 1:** MIC, MBC, MBIC_50_ and MBRC_50_ of AR extract against test bacteria.

*S. mutans* UA159	MIC_50_	MBC	MBIC_50_	MBRC_50_
(mg/mL)	1.0	8.0	4.0	8.0

*MIC* minimal inhibitory concentrations, *MBC* minimum bactericidal concentration, *MBIC* minimum biofilm inhibition concentration, *MBRC* minimum biofilm reduction concentration.

### Effects of AR extract on *S. mutans* biofilm structure

**Fig. 3:**
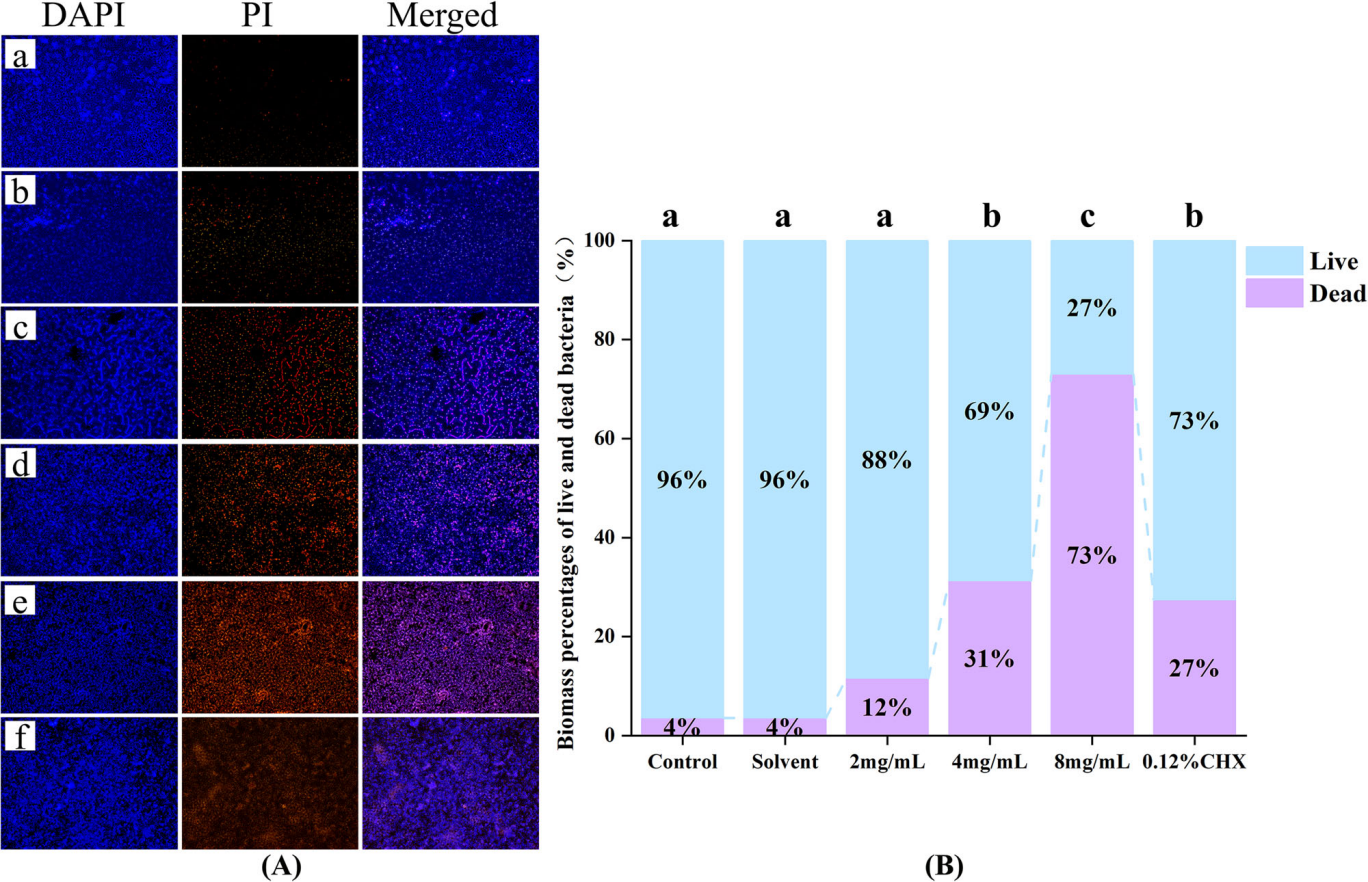
The effect of AR extract on *S. mutans*. **A** a–f shows typical fluorescence microscopy image acquisition of live, dead and merged in preformed biofilms of S. mutans after 24 h of intervention (×100). a negative control group; (b) solvent control group; (c) 2 mg/mL AR extract; (d) 4 mg/mL AR extract; (e) 8 mg/mL AR extract; (f) 0.12% CHX positive control group (n = 3). **B** The percentage of area occupied by live and dead bacteria was calculated for each group based on three randomly selected images. Dissimilar letters indicate significantly different values (*P* < 0.05).

Referring to the MBIC_50_ of AR extract, we used a 1/2 MBIC_50_, MBIC_50_ and MBIC_50_ (concentration range of 8-2 mg/mL) for live and dead bacteria staining. The formed biofilms were fluorescently stained using the DAPI/PI staining method to distinguish the live and dead bacteria in them, and the effect of AR extract on the biofilm structure of *S. mutans*, as shown in Fig. 3A(a–f). The blue fluorescence indicates the overall number of bacteria, whereas the pink fluorescence indicates the number of dead bacteria. Figure 3A contains three randomly selected fields of view. The ratio of live and dead bacteria in the area was calculated, and the Result is shown in Fig. 3B and Table 2. After 24 h of *S. mutans* biofilm intervention by AR extract, the percentage of live bacteria area decreased gradually with increasing drug concentration, where 8 mg/mL AR extract was greater than 0.12% CHX (*P* < 0.05) and 4 mg/mL AR extract was slightly greater than 0.12% CHX (no statistical difference). Even while the 2 mg/mL AR extract group had a lower percentage of living bacteria than the 0.12% CHX group, it was still possible to see that the biofilm was loose and distributed, showing a trend of destruction. The biofilms formed in the negative control and solvent groups were dense and concentrated and contained live bacteria in a large area. The results showed that the 1% DMSO (solvent control) had no impact on biomass. These findings showed that the AR extract effectively inhibited the growth of *S. mutans* biofilm. We first captured images containing fluorescence signals of live and dead bacterial samples using a fluorescence imaging system. Then, we normalized these images and subsequently utilized ImageJ software to separate the bacteria from the background. After segmentation, we extracted features such as fluorescent area and fluorescent intensity of the live and dead bacterial regions based on the characteristics of the fluorescence probe staining. The features, where blue fluorescence indicates live bacteria, red fluorescence denotes dead bacteria, and pink fluorescence signifies dead bacteria, were then used to classify the live and dead bacteria. Furthermore, we examined the ratio of live bacterial coverage to the total bacterial population.

**Table 2:** Biomass of live and dead bacteria (%).

Bacteria	Control	Solvent	2 mg/mL	4 mg/mL	8 mg/mL	0.12% CHX
Dead	3.54 ± 0.80^a^	3.54 ± 0.25^a^	11.58 ± 1.67^a^	31.49 ± 6.08^b^	73.06 ± 4.00^c^	27.02 ± 2.81^b^
Live	96.46 ± 0.80^a^	96.46 ± 0.25^a^	88.42 ± 1.67^a^	68.51 ± 6.08^b^	26.94 ± 4.00^c^	72.98 ± 2.81^b^

Data are expressed as mean ± standard error (*n* = 3), Dissimilar letters indicate significantly different values (*P* < 0.05).

### AR extract can reduce the adhesion of *S. mutans* to saliva-coated HAP

**Fig. 4:**
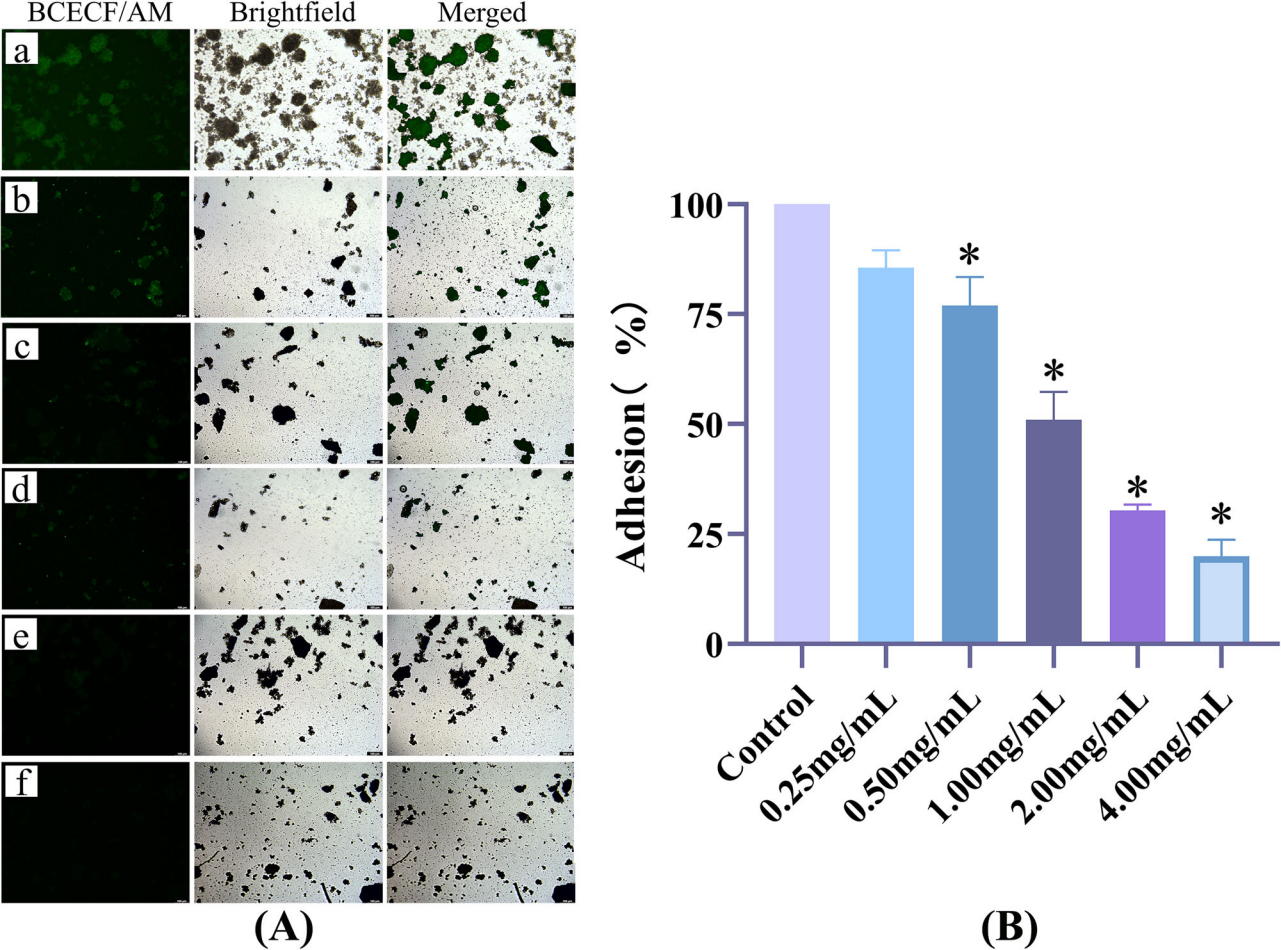
The effect of AR extract on *S. mutans* adhesion to salivary coated HAP. **A** a–f shows the image acquisition of bacterial adherence to saliva-coated HAP under fluorescence microscopy (×100). The green fluorescence is BCECF/AM-labeled S. mutans. In the 1, 2, and 4 mg/mL AR extract intervention groups, the HAP clusters were visible in the bright field, but there was little or very little fluorescence apparent in the fluorescence field. a Negative control group; (b) 0.25 mg/mL; (c) 0.5 mg/mL; (d) 1 mg/mL; (e) 2 mg/mL; (f) 4 mg/mL. **B** In comparison to the S. mutans group, the adhesion rate of S. mutans on the surface of saliva-coated HAP was considerably lower following AR extract intervention (*P < 0.001). Bacterial adherence rate = fluorescence value of adherent bacteria/positive control group ×100%.

One of the key elements in the development of dental caries is *S. mutans*’ capacity to cling to and colonize the tooth surface. Plaque is created when planktonic bacteria from the oral cavity stick to the surface of tooth enamel. In this study, *S. mutans* was fluorescently labeled using a BCECF/AM probe, and the effect of AR extract on the bacteria’s ability to adhere to saliva-coated HAP was assessed. Figure 4A demonstrates how the fluorescence intensity on the surface of HAP gradually decreased with increasing AR extract concentration. The fluorescence intensity of adherent bacteria around HAP was significantly reduced in the 1.0 mg/mL AR extract groups, whereas the green fluorescence around HAP was barely perceptible in the 2 and 4 mg/mL AR extract groups. The results in Fig. 4B and Table 3 also demonstrate that the concentration of the AR extract was inversely correlated with *S. mutans*’s capacity to adhere to saliva-coated HAP. At 0.5 mg/mL, the AR extract inhibited bacterial adhesion (*P* < 0.001), and at 1.0 mg/mL, it reduced *S. mutans* bacterial adhesion to 50.93% (*P* < 0.001), meaning that at least half of the bacteria were no longer being able to adhere to saliva-coated HAP.

**Table 3:** Adhesion rate of *S. mutans* to salivary coated HAP (%).

Group	UA159	0.25 mg/mL	0.5 mg/mL	1.0 mg/mL	2.0 mg/mL	4.0 mg/mL
Data	100.00 ± 4.19	85.52 ± 4.05	77.00 ± 6.45^*^	50.93 ± 6.36^*^	30.39 ± 1.34^*^	19.92 ± 3.80^*^

Data are expressed as mean ± standard error (*n* = 3), *P < 0.001.

### Evaluation of the dental caries in rat model

#### AR extract significantly reduces the number of salivary *S. mutans* in the oral cavity of rats

**Fig. 5:**
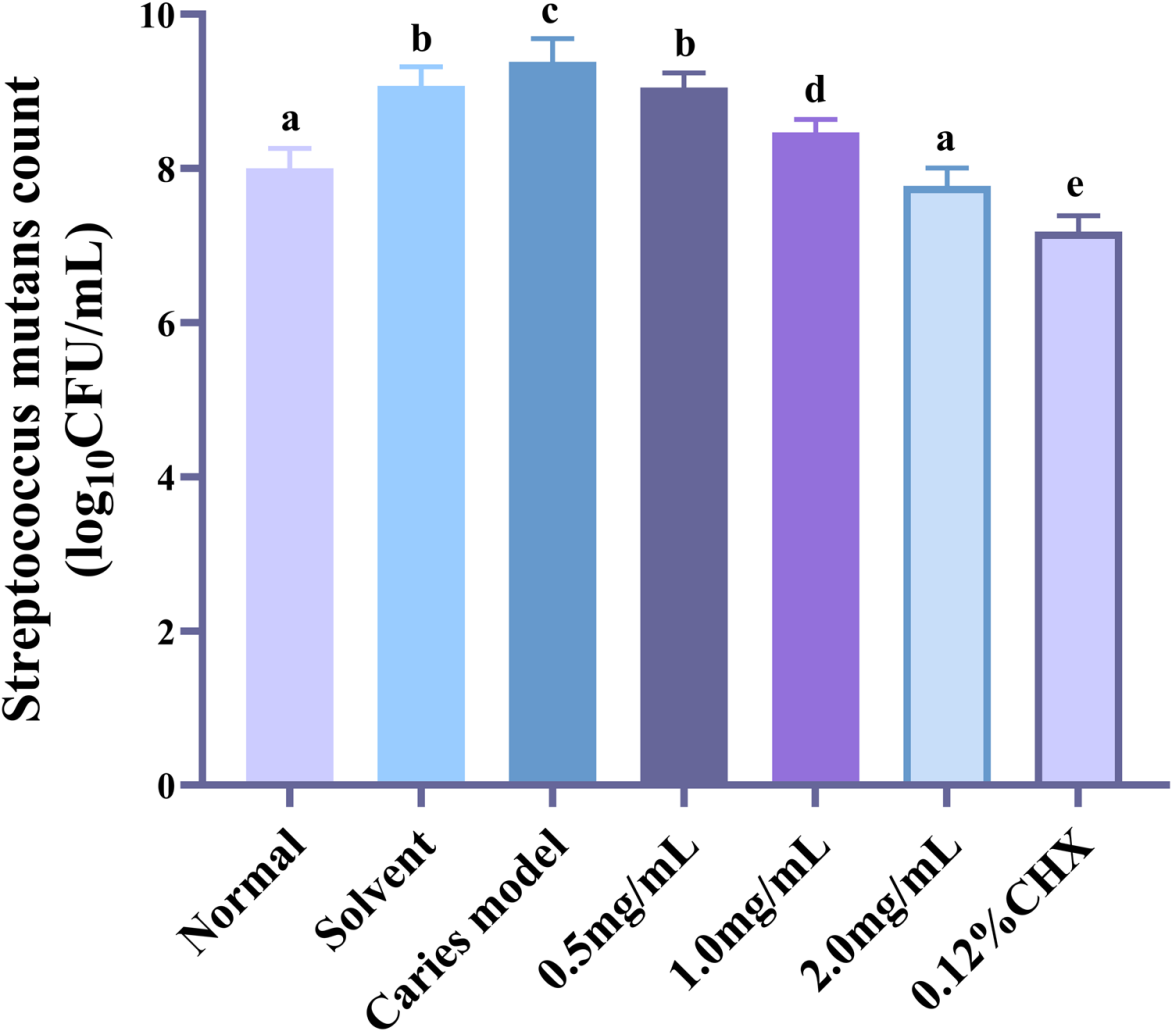
Effect of AR extract on S. mutans in rat saliva. S. mutans colonies in rat saliva were counted at the end of the experiment (*n* = 6), and the data were expressed as mean ± standard error. Dissimilar letters indicate significantly different values (*P* < 0.05).

This study used a rat caries model for in vivo tests to further explore the antibacterial effect of AR extract on dental caries in light of its antibacterial action on *S. mutans* and its biofilm in vitro. We discovered that AR extract could significantly lower the number of *S. mutans* in the oral cavity by counting the *S. mutans* in the rats’ saliva. Figure 5 and Table 4 show that there were considerably less *S. mutans* in the oral cavities of rats in the 0.5 mg/mL, 1 mg/mL, 2 mg/mL AR extract, and 0.12% CHX groups compared to the caries model group (*P* < 0.01). All of the aforementioned findings suggested that topical application of AR extract could successfully lessen *S. mutans* colonization and proliferation in the oral cavity of rats.

**Table 4:** *S. mutans* counts from rat saliva (log_10_ CFU/mL).

Group	Normal	Solvent	Caries model	0.5 mg/mL	1.0 mg/mL	2.0 mg/mL	0.12% CHX
Data	8.00 ± 0.26^a^	9.07 ± 0.25^b^	9.38 ± 0.30^c^	9.05 ± 0.19^b^	8.47 ± 0.17^d^	7.77 ± 0.23^a^	7.18 ± 0.20^e^

Dissimilar letters indicate significantly different values (*P* < 0.05).

#### Keyes scoring and X-ray manifestations

**Fig. 6:**
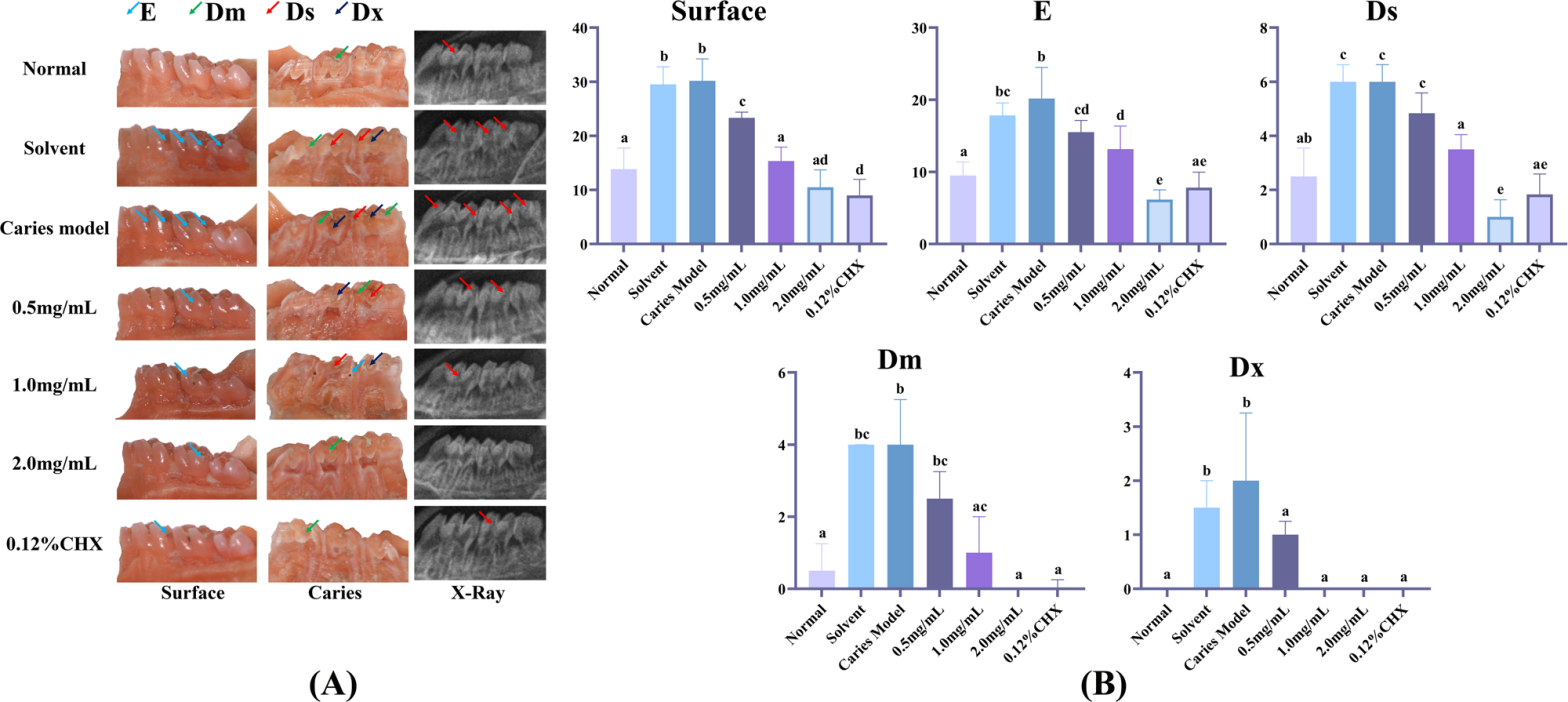
Inhibition of caries occurrence and severity in vivo by topical application of AR extract. **A** Smooth surface and sagittal section of the rat mandibular dentition under the stereomicroscope, arrows represent carious lesions, different color arrows represent different degrees of carious lesions. X-ray photography: X-rays of rat molars taken at the end of the AR extract treatment, where red arrows are marked as carious lesions. **B** The Keyes score in the molar teeth of SPF-SD rats. Surface: Surface caries; E: enamel caries; Ds: dentin exposed; Dm: three-fourths of the dentin affected; Dx: 4/3 or full thickness of dentin depth; (Surface, E, Ds) Data are expressed as mean ± standard error (*n* = 6); (Dm, Dx). Data are expressed as mean ± standard error (*n* = 6), Dissimilar letters indicate significantly different values (*P* < 0.05).

We used a well-established animal model of dental caries to test whether topical treatment of AR extract can prevent the development and severity of dental caries in vivo. The molar dental caries of rats was observed using body vision microscopy and X-ray photography, the findings were displayed in Fig. 6A, while the above results were scored by the Keyes method, as shown in Fig. 6B and Table 5. In the caries model group and solvent control group, widespread pit, fissure, and smooth surface caries lesions were identified, as represented by arrows. In the AR extract treatment group, the Keyes scores of surfaces, enamel only (E), and dentin exposed (Ds) levels in the 0.5 mg/mL AR extract treatment group were significantly different from those in the caries model group (*P* < 0.01). The Keyes scores at the surface, E, Ds, 3/4 of affected dentin (Dm), and all dentin affected (Dx) levels in the 1 mg/mL and 2 mg/mL AR extract treatment groups were significantly different from those of the caries model group (*P* < 0.001), and the results were similar to the 0.12% CHX treatment group. According to the aforementioned findings, topical applications of 1 mg/mL and 2 mg/mL AR extract effectively reduced the frequency and severity of caries lesions at all levels on smooth and pitted surfaces. The number of smooth surfaces, E, and Ds caries lesions can be decreased with topical application of 0.5 mg/mL AR extract, but the depth of deep caries developed cannot be decreased. According to the aforementioned findings, topical applications of 1 mg/mL and 2 mg/mL AR extract effectively reduced the frequency and severity of caries lesions at all levels on smooth and pitted surfaces. The number of smooth surfaces, E and Ds caries lesions can be decreased with topical application of 0.5 mg/mL AR extract, but the depth of deep caries developed cannot be decreased.

**Table 5:** Keyes caries scores.

Group	Surface	E	Ds	Dm	Dx
Normal	13.83 ± 3.92^a^	9.50 ± 1.87^a^	2.50 ± 1.05^a,b^	0.50(0.00,1.25) ^a^	0.00(0.00,0.00)^a^
Solvent	29.50 ± 3.27^b^	17.83 ± 1.72^b,c^	5.56 ± 1.03^c^	4.00(2.75,4.00) ^b,c^	1.50(2.00,1.00)^b^
Caries model	30.17 ± 4.07^b^	20.17 ± 4.31^b^	5.67 ± 1.03^c^	4.00(3.75,5.25) ^b^	2.00(2.00,3.25)^b^
0.5 mg/mL	23.33 ± 1.03^c^	15.50 ± 1.64 ^c,d^	4.83 ± 0.75^c^	2.50(2.00,3.25) ^b,c^	1.00(0.75,1.25)^a^
1.0 mg/mL	15.33 ± 2.58^a^	13.17 ± 3.19^d^	3.50 ± 0.55^a^	1.00(1.00,2.00) ^a,c^	0.00(0.00,0.00)^a^
2.0 mg/mL	10.50 ± 3.21^a,d^	6.17 ± 1.33^e^	1.33 ± 1.03^e^	0.00(0.00,0.00) ^a^	0.00(0.00,0.00)^a^
0.12%CHX	9.00 ± 2.97^d^	7.83 ± 2.14^a,e^	1.83 ± 0.75^a,e^	0.00(0.00,0.25) ^a^	0.00(0.00,0.00)^a^

E, enamel caries; Ds, dentin exposed; Dm, 4/3 of the dentin affected; Dx, 4/3 or full thickness of dentin depth; (Surface, E, Ds) Data are expressed as mean ± standard error (*n* = 6); (Dm, Dx) Data are expressed as Media (IQR), (*n* = 6), Dissimilar letters indicate significantly different values (*P* < 0.05).

#### Observations by CLSM (Confocal Laser Scanning Microscope)

**Fig. 7:**
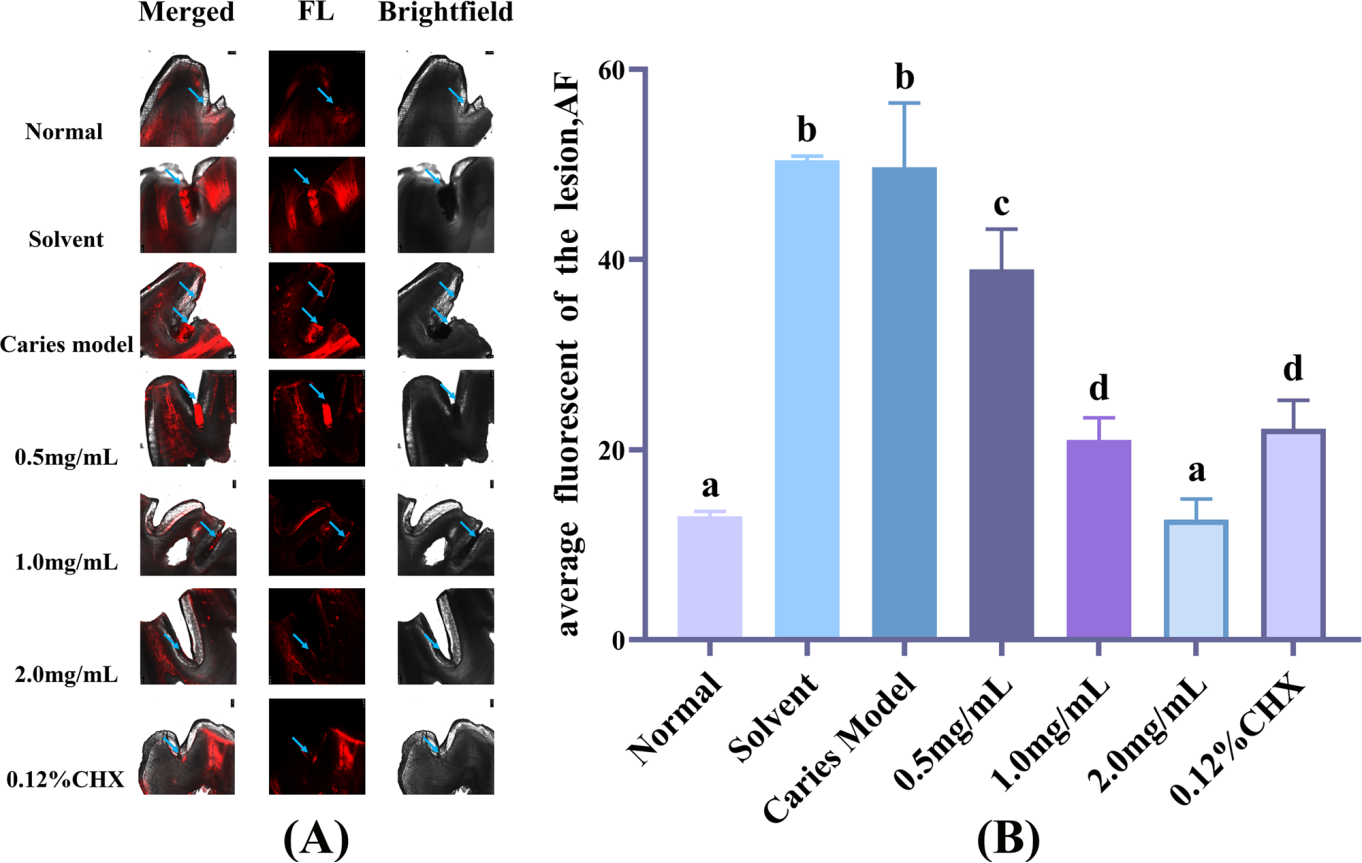
Enamel imaging using CLSM following AR extract treatment. **A** Representative CLSM images after AR extract treatment, blue arrows indicate the increase in enamel surface porosity due to caries damage. **B** AF is the average amount of fluorescence per unit area of lesion area after fluorescence staining, data are expressed as mean ± standard error (*n* = 4). Different superscript letters indicate significant differences. *P* < 0.05.

Enamel demineralization causes the surface pores to widen in the early stages of caries development, which allows the fluorescent staining solution to enter the enamel pores. Under CLSM, the fluorescence is visible, and the more intense the fluorescence, the more severe the demineralization occurrence. Figure 7 and Table 6 show the results of our use of CLSM to observe the onset and severity of dental caries in rats. We discovered that the enamel was continuous and regular in the 2.0 mg/mL AR extract treatment group and the 0.12% CHX treatment group. No obvious enamel fissures were found, and the fluorescence intensity was significantly lower than it was in the caries model group. In the 0.5 and 1.0 mg/mL AR extract treatment groups, the fluorescence intensity and the number of cracks in the enamel were between the caries model group and the 2.0 mg/mL AR extract treatment group.

**Table 6:** The mean fluorescence per unit area of caries area in different groups of rat enamel after fluorescent staining under CLSM.

Group	Normal	Solvent	Caries model	0.5 mg/mL	1.0 mg/mL	2.0 mg/mL	0.12% CHX
Data	13.00 ± 0.50^a^	50.41 ± 0.47^b^	49.71 ± 6.75^b^	38.95 ± 4.25^c^	21.01 ± 2.32^d^	12.64 ± 2.16^a^	22.17 ± 3.01^d^

Data are expressed as mean ± standard error (*n* = 4), different superscript letters indicate significant differences, *P* < 0.05.

#### Good biocompatibility of AR extract in rats

**Fig. 8:**
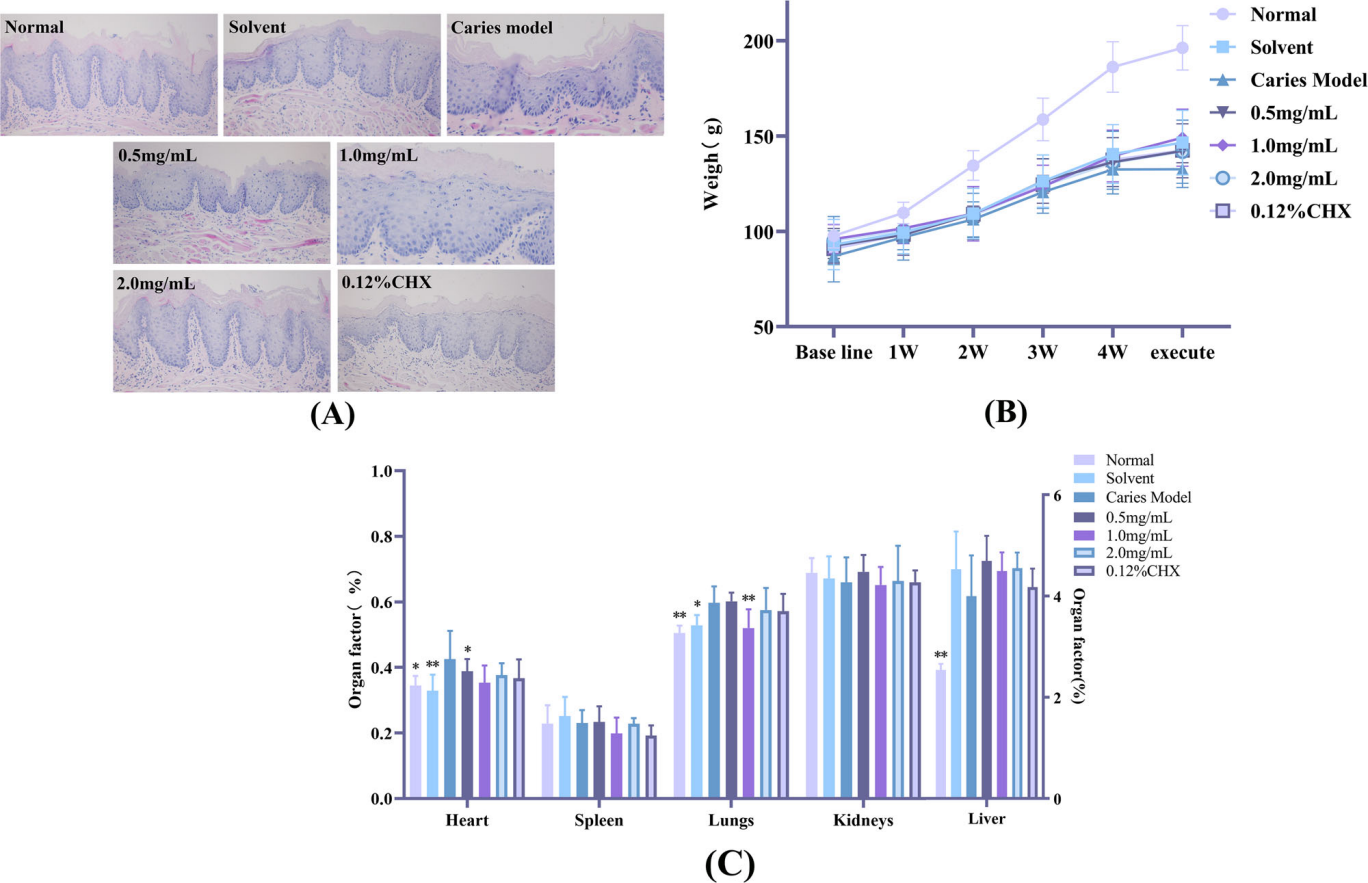
Evaluation of the safety of AR extract. **A** HE staining pathological sections of buccal mucosa; (**B**) Weight of rats during the intervention; (**C**) Main organ coefficient of rats, *P < 0.05, **P < 0.01.

By measuring body weight, examining the oral mucosa’s histology, and using organ coefficient, we further assessed the biocompatibility of AR extract in vivo. All of the rat groups survived the whole trial in good health, with normal vitality and no dead rats. During the treatment period, the body weight of the rats increased continuously, and we found that except for the normal group fed with normal food, the body weight of each treatment group fed with Diet 2000 food and 5% sucrose water increased steadily, and there was no significant difference between the groups, and the results are shown in Fig. 8B. To demonstrate the safety of AR extract treatment on oral mucosal tissues, we performed HE staining on buccal mucosal tissues, and the results are shown in Fig. 8A. The epithelial layer of the oral mucosa was intact in all groups of rats, the stratum corneum was smooth and coloration was uniform, the basal cell layer was continuous and had a normal shape, the rete pegs extended toward the basal layer, and no pathological changes like erosion, ulceration, or inflammation were seen. In addition, we collected and weighed the heart, liver, spleen, lungs, and kidneys to determine the organ coefficient (organ coefficient = organ weight / body weight × 100%), and the results are shown in Table 7 and Fig. 8C. We discovered that the kidney coefficient did not differ significantly from the caries model group, and the liver coefficient did differ slightly between the treatment groups and the caries model group, but the difference was not statistically significant. All of these findings show that using AR extract to prevent and treat dental caries progression is a biocompatible strategy.

**Table 7:** Main organ coefficient (%).

Group	Heart	Spleen	Lungs	Kidneys	Liver
Normal	0.35 ± 0.03^*^	0.23 ± 0.06	0.51 ± 0.05^**^	0.69 ± 0.05	2.54 ± 0.12^**^
Solvent	0.33 ± 0.05^**^	0.25 ± 0.06	0.53 ± 0.05^*^	0.67 ± 0.07	4.53 ± 0.75
Caries model	0.43 ± 0.09	0.23 ± 0.04	0.60 ± 0.03	0.66 ± 0.08	4.00 ± 0.80
0.5 mg/mL	0.39 ± 0.04^*^	0.23 ± 0.05	0.60 ± 0.06	0.69 ± 0.05	2.54 ± 0.12^*^
1.0 mg/mL	0.35 ± 0.05	0.20 ± 0.05	0.52 ± 0.07^**^	0.65 ± 0.06	4.49 ± 0.37
2.0 mg/mL	0.38 ± 0.04	0.23 ± 0.02	0.58 ± 0.06	0.66 ± 0.11	4.55 ± 0.31
0.12% CHX	0.37 ± 0.06	0.19 ± 0.03	0.57 ± 0.03	0.66 ± 0.04	4.18 ± 0.37

Data are expressed as mean ± standard error (*n* = 6), *P < 0.05, ** *P* < 0.01.

## Discussion

The AR, a naturally occurring plant with significant medicinal value, are abundant in a number of secondary metabolites, such as naphthoquinone pigments, meroterpenoids, and arnebinols [37]. Ethanol can be used as a solvent to extract a number of active components, including shikonins and hydroxynaphthoquinone pigments [38].

Shikonins, a collective word for a variety of naturally occurring naphthoquinone compounds that can be extracted using ethanol, are one of them. According to ongoing research [39], at least 10 different naphthoquinone components have been found in the AR. Damianakos et al. found that different chemical components of the *Arnebia euchroma* (Royle) Johnst. have different antibacterial abilities against different types of bacteria, and Shikonin, Acetyl Shikonin, β-Hydroxyisovalerylshikonin, and Isobutyryl shikonin were the most active antibacterial components. Shikonin was more efficient against gram-positive bacteria and inhibited five common pathogenic microorganisms in a test tube, including *Escherichia coli* (*E. coli*), *Staphylococcus aureus* (*S. aureus*), and *Streptococcus agalactiae* (*S. agalactiae*), for gram-positive bacteria, the inhibitory impact was more potent [21]. Other components of AR included butyryl alkannin and isovalerylshikonin, both of which demonstrated excellent antibacterial potential against highly drug-resistant *Enterococcus faecalis* (*E. faecalis*) (MIC = 3.16–6.26 μg/mL) and drug-resistant *S. aureus* (MIC = 16 μg/mL) respectively [40].

The main antibacterial mechanism of the *Arnebia euchroma* (Royle) Johnst. extract is that it alters the morphology and structure of bacteria [41], leading to the destruction of their cell walls and membranes, thereby altering cell permeability. Shikonin possesses the ability to disrupt the physiological properties regulated by N-acyl-L-homoserine lactones in the Gram-negative bacterium Chromobacterium violaceum, thus inducing quorum sensing (QS) inhibition. Furthermore, acetyl-shikonins are effective only in inhibiting biofilm formation. This demonstrates the diverse functions of shikonin and acetyl-shikonins in their interactions with bacteria [42]. Quorum sensing is a communication process in bacteria that involves the secretion of autoinducer molecules, thereby facilitating the regulation of biofilm formation through the synthesis of extracellular polysaccharides and intercellular polysaccharide adhesins. This mechanism plays a crucial role in microbial communities by influencing the interactions among bacterial populations. Moreover, *S. mutans* utilizes quorum sensing to competitively thrive within the oral cavity and to exert pathogenic effects. By responding to environmental cues through quorum sensing, S. mutans can effectively adapt to and manipulate its ecological niche in the oral microbiome [43]. One of the potential anti-biofilm mechanisms of Shikonin is its inhibition of *S. mutans*’s quorum sensing. Furthermore, Shikonin demonstrated inhibitory effects on *Listeria monocytogenes* biofilm formation, altered biofilm morphology, and attenuated adhesion. Additionally, it downregulated the expression of virulence factors associated with *Listeria monocytogenes* [44]. In this experiment, ethanol was used as the extraction solvent to specifically extract the naphthoquinones, the active ingredients from AR, for the purpose of evaluating their anti-caries properties in both in vivo and in vitro studies. Neuraminidase (NA), a significant factor in causing infections, is widely known for its involvement in various biological processes and is deemed as a major virulence factor. Zawawy et al. [45] found that the NA inhibitor CS-MC-GIC-4 completely inhibits the expression of NA in *Candida albicans*, potentially providing a new approach to preventing dental/oral infections. Twelve compounds, such as Isovalerylalkannin, β, β-Dimethylacrylalkannin, and Deoxyshikonin, were isolated and identified from the alcohol extract of *Arnebia euchroma* (Royle) Johnst. These compounds showed effective inhibitory activity against bacterial NA [46]. Our initial study shows that extracts inhibit both planktonic and biofilm forms of bacteria at different concentrations, ranging from twice the MIC to three lower levels. This inhibition affects the production of acid and water-insoluble polysaccharides [20].

It is well known that biofilms mediated by cariogenic bacteria play a crucial role in the progression of dental caries. In the early 1960s, *S. mutans* was identified as the primary pathogenic bacteria of dental caries [47]. Among the complex oral microbiome, *S. mutans* are particularly prominent in the pathophysiology of dental caries, as this bacterium is the main producer of the exopolysaccharide (EPS) matrix [48], an important component of cariogenic biofilms. EPS can promote the adhesion, accumulation, and aggregation of cariogenic bacteria on the tooth surface, further promote the formation of spatially heterogeneous bacterial niches, form complex three-dimensional structures, protect biofilms, and regulate the pathogenesis of dental caries. Therefore, it has been demonstrated that removing or lowering the amount or virulence of *S. mutans* in the oral microenvironment prevents or slows the development and/or progression of dental caries [49]. Fluoride, CHX, quaternary ammonium salts, and antimicrobial peptides (AMPs) are currently used to control oral *S. mutans*. Among these, CHX was one of the earliest antibiotics proposed for the prevention and treatment of dental caries and has proven to be the most effective [50]. Due to its strong bacteriostatic capacity, CHX is frequently formulated into mouthwash (0.06–0.2%) and is commonly used in clinics and households to remove dental plaque [8]. In addition, researchers frequently utilize it as the gold standard to evaluate the anti-cariogenic efficacy of the investigated medicines, with 0.12% CHX being the most prevalent [9]. However, excessive CHX usage also results in oral microecological disorder and the overgrowth of drug-resistant bacteria or fungus. In the recent past, the research prospects for the active components of natural medicines have expanded because of their wide variety, amazing effectiveness, and good biological safety [51]. In addition, our previous study showed that the ethanolic extract of AR inhibited the growth, acid production, water-insoluble polysaccharide production and adhesion of the main cariogenic bacteria in the oral cavity [20], which gave us a preliminary understanding of the anti-caries ability of AR extract. Therefore, in this study, we evaluated the effective components of the AR extracts on inhibiting the biofilm activity of *S. mutans* and their ability to adhere to HAP, as well as its caries prevention effectiveness and biosafety in the rat caries model.

First, in vitro investigations determined the MIC_50_ (1 mg/mL) and MBC (8 mg/mL) of AR extract against planktonic *S. mutans* and proved that it inhibits the growth of *S. mutans*. As the primary pathogenic form of *S. mutans*, dental plaque is significantly more resistant to external mechanical and pharmacological resistance than the planktonic condition [52]. The impact of AR extract on *S. mutans* biofilm MBIC_50_ (4 mg/mL) and MBRC_50_ (8 mg/mL) was then evaluated. The MBIC_50_ and MBRC_50_ of AR extract to the *S. mutans* biofilm we determined are higher than the MIC_50_, mainly due to the different culture methods of *S. mutans* used in the above method, where the former method enables *S. mutans* to form a stable and dense plaque biofilm structure, thus causing the difference in these results. For the determination of MBRC_50_, a static biofilm growth assay was used in this experiment, which is particularly effective in examining the early events of biofilm formation, enabling the detection of biofilm formation and changes in a short period of time [53]. In contrast, the bacteria in MIC’s experiments were taken to grow in a continuous flow environment, a process in which the AR extract was able to fully engage with *S. mutans* cells and exert antimicrobial effects. Plaque biofilm formation mainly involves three stages: acquired film formation on tooth surfaces; bacterial adhesion; and biofilm maturation [54]. Therefore, anti-caries agents should be effective in inhibiting the formation of new biofilms and reducing the viability of existing biofilms, rather than only inhibiting the growth of planktonic bacteria [55]. The AR extract showed a good inhibitory effect on *S. mutans* biofilm formation. We observed the effect of MBRC_50_ And the following two concentrations AR extract on established mature *S. mutans* biofilms under stained fluorescence microscopy. The mature biofilm was encapsulated by the extracellular matrix, which is difficult to remove. Even after treatment with CHX at a clinical concentration of 0.12% for 24 h, the bacterial cells in the biofilm could not be completely removed or killed. It was observed that the structure of the biofilm treated with AR extract was loose and disordered, and the number of dead bacteria emitting red fluorescence in the biofilm of *S. mutans* treated with AR extract increased significantly. As the concentration of AR extract increased, the number of dead bacteria increased, and the amount of biofilm decreased. As for the possible mechanism by which AR extract remove mature *S. mutans* biofilms, based on our results, we hypothesized that 2.0 mg/mL AR extract might be able to enter small channels in biofilms, penetrate the thick extracellular matrix, kill cells, and eventually disrupt and lyse the structure of *S. mutans* biofilms. However, the higher concentration of AR extract caused the death of bacteria in the biofilm and the structural collapse. Bacterial adhesion is the most important stage and can be disrupted using appropriate methods. The adhesion of *S. mutans* includes both sucrose-independent and sucrose-dependent modes [56]. Therefore, this study tested the ability of bacteria to adhere to HAP after incubation with AR extract. In this study, AR extract inhibited the adhesion of *S. mutans* to HAP, among them, 1 mg/mL made 50% of the bacteria lose the ability to adhere. which suggests that AR extract may inhibit bacterial adhesion to the tooth surface. All of the aforementioned data suggested that AR extract not only decreased the area of *S. mutans* biofilm, but also damaged its biofilm structure, reduced its vitality, and diminished its adhesion capacity.

In *vivo*, we observed that AR extract has strong antibacterial, anti-adhesion and anti-biofilm formation ability against *S. mutans* and its biofilm, but it does not mean that it can play the effect role in vivo. The anti-caries drugs actually used in clinic do not completely kill cariogenic bacteria in the mouth, but control the activity and number of cariogenic bacteria at a low level [57]. At the same time, saliva contains some antibacterial substances, and the use of drugs may also have a positive or negative impact on the antibacterial ability of saliva [58]. Therefore, this study further tested the anti-caries activity of AR extract in a rat dental caries model. This study examined the anti-caries efficacy of AR extract in a rat model of dental caries. This study assessed the in vivo anti-caries safety and efficacy of AR extract using a modified Keyes rat dental caries model [59], and the Diet 2000# cariogenic formula with a high sucrose content and high viscosity [60]. To confirm the anti-caries effect of AR extract, we used the Keyes score to evaluate the degree of caries in rats. The Keyes scoring system is a classic caries scoring system that can evaluate the degree of caries in rats [35]. Molars are more sensitive to Diet 2000# and Keyes score, which is strongly supported by the corresponding images (Fig. 6A). The results of the Keyes score confirmed that both AR extract and CHX had better caries-preventing effects than the DDW group on both the smooth surface and groove site. 1 mg/mL AR extract, 2 mg/mL AR extract, and 0.12% CHX can significantly reduce the quantity of superficial and central caries and inhibit the progression of caries into deeper areas. Although the effect of AR extract at a dosage of 0.5 mg/mL was not as pronounced as that of the high-concentration group, it was still able to lower the incidence of superficial caries to some extent. Regarding enamel caries, the 2 mg/mL AR extract group had an anti-caries effect comparable to that of the CHX group. At the same time, X-Ray technology was utilized to assess the severity of carious lesions [61], and CLSM was used to observe the enamel’s potential to demineralize or promote its remineralization, both also showed that the AR extract had a significant anti-caries effect in rat with dental caries. The results of colony counting showed that the AR extract and CHX groups had reduced numbers of *S. mutans* compared to the DDW group, and both showed inhibition of *S. mutans*, but were weaker than the CHX group. The data presented above demonstrated that AR extract greatly decreased the cariogenic virulence of *S. mutans* but did not significantly disturb the microecological balance of oral flora. In addition, in vivo experiments can also help to judge drug safety [62]. Building on previous research, the observation of hepatoprotective activity when oral arginine (AR) extracts were taken, their effects on platelet production and coagulation, as well as their capability to improve and modify liver function damage, has substantiated this claim. In particular, a dosage of 3.5g kg-1day-1 was found to yield the most optimal effects [63]. The main components in AR extracts, including naphthoquinone 6, steroid 18, and triterpenoid 19, demonstrated moderate inhibitory effects on ATP-citrate lyase and protein tyrosine phosphatase 1B, while not exhibiting obvious cytotoxicity [64]. In our experiment, there was no significant difference in body weight between rats fed cariogenic diets, and no pathological alterations (erosion, ulcer, etc.) were observed in the oral mucosa, indicating that AR extract (0.5–2 mg/mL) did not have a significant toxicity effect in vivo. All of the aforementioned data suggested that AR extract might be a source of a novel natural topical anti-caries agent that prevents the incidence and development of dental caries without altering the microflora balance.

However, it is well known that a single carious bacterium does not cause dental caries, nor is it a static process. There are various interactions between the complex plaque biofilm and planktonic bacteria in the oral cavity. Whether AR extract can reduce the number or toxicity of cariogenic bacteria to maintain the stability of oral microecology needs further research and verification. The results of the medium and high concentration AR extract intervention groups were similar to those of the CHX group, according to the Keyes scoring results. In the meantime, the low-concentration AR extract intervention group can also reduce the incidence of superficial caries to a certain extent, indicating that the low-concentration AR extract intervention also has the effect of preventing caries; however, it is unclear whether it reduces the incidence of caries or slows the progression of caries, and longer experiments are required to confirm.

The widely accepted criteria for validating animal models include predictive validity, face validity, and construct validity. However, a single model is unlikely to fully replicate a human disease and may not satisfy all three criteria [65]. *S. mutans* can cause dental caries in rats, which is used to evaluate the in vivo anti-caries efficacy of AR extract, but human dental caries is caused by a combination of various cariogenic bacteria, complex diet, host factors and time of action, but also a dynamic development process. The caries model used in this study simulates the phenotypic manifestation of human dental caries, including key traits like cavitations, thereby giving it “face validity” characteristics. It replicates the disease’s etiological mechanisms such as *Streptococcus mutans’* ability to cause dental caries in the presence of sugar in both animals and humans, demonstrating “construct validity.” In addition, various in vivo studies on traditional Chinese herbal medicines have demonstrated their efficacy in preventing caries. These studies have shown that when the experimental group is subjected to consistent conditions of age, bacterial infection, and cariogenic diet, it consistently exhibits outcomes similar to the model group [32, 66, 67], thus validating its predictive validity. This aligns with the aim of “achieving a blend of diverse yet complementary models that can offer validity across the predictive, face, and construct validity criteria.” However, the dental caries model caused by *S. mutans* still has some limitations, and it is still necessary to look forward to animal model research that is more in line with the occurrence and development of human dental caries, to provide high-level evidence for the clinical application of AR extract. In addition, in future research, we also need to overcome the limitations of natural medicine, identify the natural medicine monomers with anti-caries activity in AR extract, and produce one or more mouthwashes or toothpastes containing AR for preventing dental caries.

## Conclusions

Taken as a whole, this study provided a deeper understanding of the anti-caries properties of AR and confirmed that AR extract is a highly effective caries prevention and treatment drug, which can significantly inhibit the planktonic state and biofilm activity of *S. mutans*, inhibit the formation of biofilm, and destroy its structure in vitro. Moreover, in a rat model of dental caries, AR extract strongly prevented the growth and adherence of *S. mutans* in the oral cavity of rats, as well as decreased the incidence and development of superficial and deep caries lesions while maintaining high biosafety. The above results open up new ways to make new dental materials and products for oral health that contain AR.

### Supplementary information

Table S1

Table S2

Figure S1 & S2

## Data Availability

The original contributions presented in the study are included in the article, further inquiries can be directed to the corresponding author.
