# Supplementary material for: Inhibitory and preventive effects of Arnebia euchroma (Royle) Johnst. root extract on Streptococcus mutans and dental caries in rats
Source: BDJ Open. 2024 Mar 2;10:15. doi: 10.1038/s41405-024-00196-6 (PMC10908817; doi:10.1038/s41405-024-00196-6)
Supplement: Supplementary file 3 — Figure S1 & S2 [file 41405_2024_196_MOESM3_ESM.pdf]

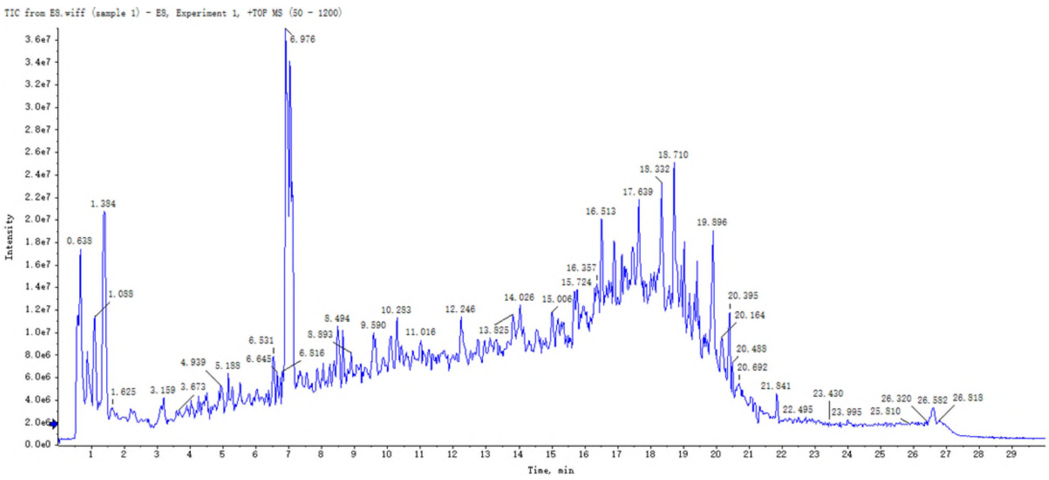

(a)

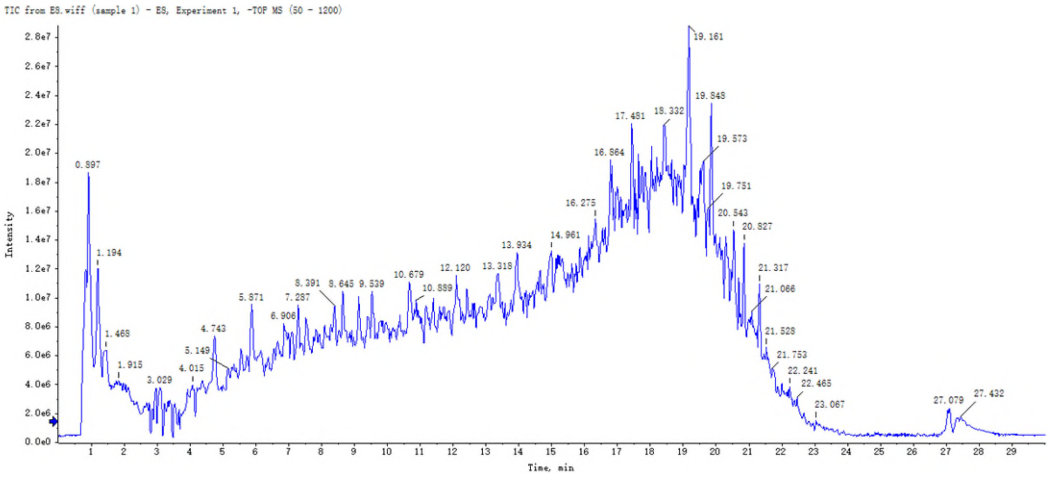

(b)

Figure S1. Chromatogram of the positive (**a**) and negative (**b**) ionization of the butanol extract of AR.

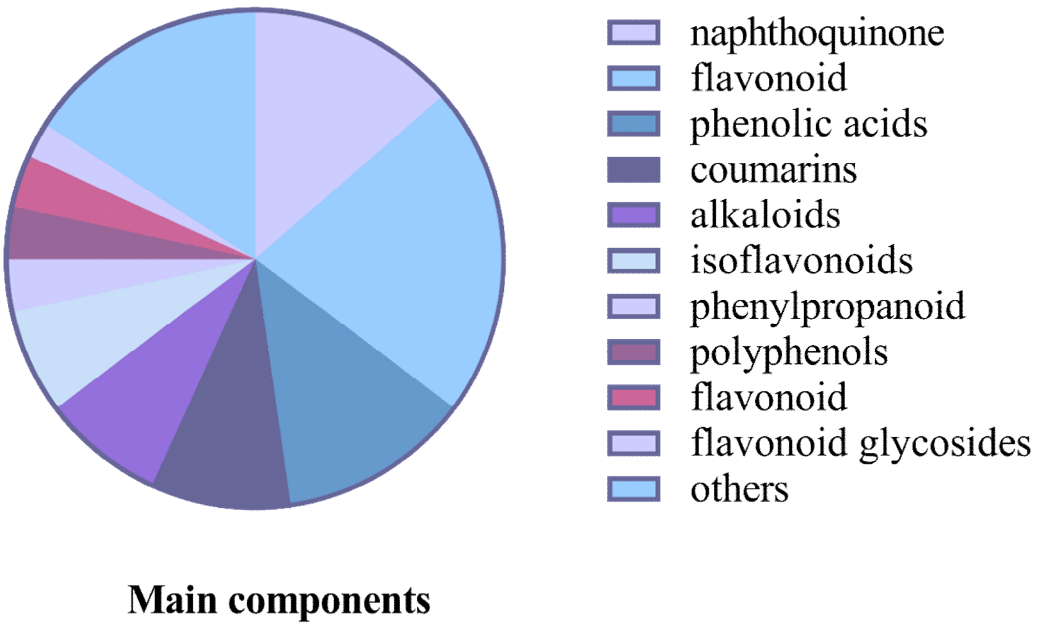

Figure S2. Classification of the main chemical compounds in the AR extract.
